# Emotional Lability as a Unique Presenting Sign of Suspected Chronic Traumatic Encephalopathy

**DOI:** 10.1155/2018/2621416

**Published:** 2018-01-14

**Authors:** Shauna H. Yuan, Sonya G. Wang

**Affiliations:** ^1^Department of Neurosciences, University of California, San Diego, La Jolla, CA 92093, USA; ^2^Department of Neurology, University of Minnesota, Minneapolis, MN 55455, USA

## Abstract

Chronic traumatic encephalopathy (CTE) is a neurodegenerative disease caused by head trauma. Diagnosis of this disease is difficult as reliable biomarkers have not been established and often this clinical entity is underappreciated with poor recognition of its clinical presentations (Lenihan and Jordan, 2015). The definitive diagnosis of CTE is determined by identification of neurofibrillary tangles in the perivascular space around the sulci in postmortem tissue (McKee et al., 2015). However, performing brain biopsies searching for neurofibrillary tangles is not a feasible option for early diagnosis. Thus, diagnosis of suspected CTE in the living has been based on clinical suspicion using proposed research criteria of clinical presentations. In addition, neuroimaging techniques have shown some promise in assisting diagnosis. Clinically, CTE is more commonly known to be associated with memory impairment and executive function disorder (Stern et al., 2013). However, here, we present two unique cases of prior professional football players where behavioral changes were the first identifying factors in clinical presentation and discuss possible neuroimaging options to help with CTE diagnosis. Because behavioral changes can be mistaken for other neuropsychological diseases, recognizing differing clinical constellations is critical to early diagnosis, early intervention, and improving patient care in suspected CTE.

## 1. Introduction

Chronic traumatic encephalopathy (CTE) has been associated with popular American contact sports, such as football, hockey, and soccer. The clinical presentation of CTE can be divided into two different groups. One group presents with changes in cognitive function, including memory and executive impairment. A second group presents with behavioral changes, including impulsivity and violent behavior prior to mood or cognitive decline [1]. This second group often can be misdiagnosed as having a psychiatric disorder and is not as easily recognized. Clinical diagnostic criteria for CTE have been proposed for research purposes but have yet to be widely disseminated for clinical purposes [1]. The utility of neuroimaging, such as MRI (magnetic resonance imaging) and PET (position emission tomography), has been evaluated to assist in early diagnosis [2]. This case report describes two unique presentations of behavioral changes as early signs of suspected CTE and provides further discussion regarding neuroimaging possibilities. This information is essential in helping to increase awareness of the differing clinical presentations of CTE.

The definitive diagnosis for CTE is neuropathological evidence of early perivascular tau deposition in the brain [3]. Tau is a microtubule binding protein, important for axonal transport. Abnormal phosphorylated tau deposition is also found in other types of neurodegenerative diseases, such as Alzheimer's disease [4]. In later stages of CTE, tau deposition appears in other regions of the brain [5, 6].

Without biomarkers, CTE continues to be a diagnostic dilemma [7]. The clinical presentation overlaps with psychiatric presentations such as depression and memory impairment. There are several proposed diagnostic criteria for research purposes [8–10] and these include history of repetitive head trauma and cognitive and/or behavioral impairment. Often symptoms associated with CTE may be ignored, and, therefore, raising awareness and recognizing the symptoms are critical. Below, we present two cases of professional American football players to illustrate behavioral and memory changes suggesting early symptoms for suspected CTE. Included in their cases are background demographic data in Table 1.

## 2. Case Presentation

### 2.1. Patient A

Patient A was a 39-year-old right-handed former professional football player. He presented to Memory Disorders Clinic in a tertiary care hospital for evaluation of memory problems. He first developed mood problems with irritability, depression, and social withdrawal about the time he retired from professional football in his early 30s. He experienced significant mood swings, sometimes laughing uncontrollably, rocking, pacing, and grumbling. He also had suicidal ideations. Later, short-term memory problems started insidiously and gradually worsened into his mid-30s. He was quick to forget, constantly repeated questions, often forgot his appointments, and misplaced objects. He had difficulty with directions and relied on GPS to get around. He would forget to pay his bills, and thus management of the finances was taken over by his caregiver. He had difficulty performing at his job, for he could not remember the tasks. He also became frustrated easily and could not multitask.

Patient A started playing football at age of 5. He played football for total of 25 years. He experienced eight concussions, in which he lost consciousness in four of them. In addition, he experienced brief “bell rung,” hitting his head and seeing stars, which occurred at least once if not more every time he played football. There is no family history of Alzheimer's disease.

His MOCA score was 20/30 and the CDR was 1. On the neuropsychological testing, patient exhibited significant deficit in multiple domains, including complex attention and executive and memory functioning (Table 2). Patient A's MRI brain was normal. Patient did not carry a psychiatric diagnosis previously. Diagnosis was major neurocognitive disorder, chronic traumatic encephalopathy, and major depressive disorder based on history and neuropsychological testing.

### 2.2. Patient B

Patient B was a 41-year-old right-handed ex-professional football player. He started feeling depressed at age of 30 and it gradually worsened over the years. He developed significant mood swings for three to four years where he cycled between feeling sad, crying, laughing, and becoming angry. He was not diagnosed or treated for mood problems, for he never sought medical help. He has lost interest in his hobbies and slowly reduced his daily activities to lying around at home.

Memory problems started at about age of 39. The memory problems had an insidious onset and gradual progression. Patient B had trouble recalling details of events. He needed to write things down; otherwise, they would not get done. He repeated questions several times a day.

His activities of daily living (ADLs), which are daily self-care activities, including eating, bathing, dressing toileting, transferring, and continence, were not entirely intact. He required reminders to take showers. Although he could dress himself, he required prompting to dress appropriately for the occasion. His instrumental ADLs (iADLs), which are activities requiring skills to successfully live independently, were affected. He was able to go shopping by himself; he could pick out the items and pay for them. His wife took over paying the bills completely, to ensure that bills were paid on time, because he forgot to pay certain bills. His driving was unaffected. He had not had any accidents and had not gotten lost. However, he used the GPS to help him get around. He continued to participate in doing household chores but typically needed assistance. For example, he could start the laundry but would forget to finish the laundry.

He started playing football since age of 13. He played 9-year professional football and 4-year college football. He played football for total of 18 years. He has had numerous events associated with seeing stars/seeing light/dazed for a few minutes during the season. He could not remember how many he had but reported having had quite a few. He did remember experiencing being knocked out four times and being escorted off the field.

His MOCA score was 23/30 and the CDR was 1. On the neuropsychological testing, patient exhibited significant deficit in multiple domains, including complex attention and executive functioning (Table 2). MRI was normal. His neuropsychological testing results and clinical history fulfilled the criteria of DSM V for major neurocognitive disorder, for he had deficits, which interfered with daily life in two different domains.

## 3. Discussion

This case report highlights an essential understanding of how CTE can develop in sports players as early as their 30s and can uniquely present with mood problems prior to memory and executive function problems.

These two cases show a similar history to other professional American sports players as both started playing football as children (Table 1). Both played in high school, college, and then professionally for approximately 10 years. Patient A started playing at an earlier age than patient B by 10 years. It is possible that patient A developed disease earlier than patient B for patient A started playing earlier. The number of years of exposure to the sport correlates with disease severity [11].

CTE is a tauopathy. NFT, tau-positive astrocytes, tau-positive cell processes that preferentially involve the cortical sulci, medial temporal lobe, diencephalon, and brainstem are observed on brain autopsy. The tau pathology is characteristically perivascular, most pronounced at the sulcal depths, and preferentially involves the superficial cortical layers. These microscopic changes accumulate over time and help increase our understanding of its pathophysiology; however, at this time, these tau changes do not help clinicians in the diagnosis of the disease.

With regard to neuroimaging, both patients retained normal brain MRIs and thus relying on brain imaging may not be helpful in early diagnosis. CTE patients can develop cavum pellucidum with brain trauma; however, this is only in a small subgroup of patients with CTE [11]. More recently, PET imaging has been considered as a potentially useful neuroimaging modality for detecting CTE pathology. Already, a PET compound, PiB (Pittsburgh Compound B), has been developed that tracks amyloid deposition in Alzheimer's disease and helps in understanding amyloid-beta pathology after head trauma [12]. In the last two years, tau specific tracers for PET have been developed, THK5317, THK5351, AV1451, and PBB3. Due to the complexity and variability of different types of tau deposits in different diseases, these tracers will need to be evaluated for efficacy in diagnosing CTE and in understanding its pathology [13]. With future understanding of the tau specific PET tracers, the patients presented in this case report, may have further confirmatory data indicating a definitive diagnosis of CTE.

For now, these two patients fall into the clinical research diagnostic criteria of mood disorder prior to memory deficits for CTE [8–10]. Their age of onset (30s–40s) is also consistent with the behavioral symptoms presenting at a younger age compared to the age of presentation for cognitive symptoms [1]. Clinical features of CTE include impairments in mood (depression, suicidality, and irritability), behavior (explosivity, violence, and impulsivity), cognition (impaired memory, executive dysfunction, and diminished concentration), and motor functioning (parkinsonism, dysarthria, gait changes, and weakness). A longitudinal study of children in higher risk concussion sports and their development into professional sports players would be most beneficial in identifying and describing CTE. At this present time, CTE is often underrecognized or misdiagnosed. It is crucial for clinicians to be particularly aware of the varying presentations of CTE, especially when patients initially complain of emotional lability or behavioral symptoms.

## Figures and Tables

**Table 1:** Patient clinical information. Patient clinical information is compared.

	Patient	
	A	B
Age	39	41
Onset	mid-30s	30
History of concussion	yes	yes
Age of playing contact sports	5	13
Childhood/premorbid psychiatric history	no	no
Family history of Alzheimer's disease or other dementia	no	no

**Table 2:** Neuropsychological testing results. Patient neuropsychological testing showing impaired memory and cognitive functions in multiple domains. *T* score of 50 is average. One standard deviation is 10 points. For example, one standard deviation below is 40.

Test	Patient	
	A	B
	*T* score	*T* score
*Complex attention*		

WAIS IV digit span	30	47
WAIS IV arithmetic	37	43
WAIS IV letter number sequencing	37	
WAIS IV coding	27	53
WAIS IV symbol search	27	50
WAIS IV cancellation	30	37
DKEFS trails-visual scanning	30	50
DKEFS trails-number sequencing	27	50
DKEFS trails-letter sequencing	20	57
CATA commission errors		21
CPT omission errors		26
CPT commission errors		31

Speech sounds perception	43	40
Seashore rhythm	25	41

*Visual-perceptual and motor/visual-motor functioning*		

WAIS-IV block design	53	43
WAIS-IV visual puzzles	37	40
WAIS-IV matrix reasoning	50	63
DKEFS tower test	40	70

*Executive functioning*		

Verbal fluency (FAS)	32	53
Trails B	37	See TN/LS
Category test	38	36
WAIS-IV similarities	40	40
DKEFS trails number/letter switching	See Trails B	57
DKEFS letter fluency	37	53
DKEFS category fluency	37	50
DKEFS switching: total correct	43	37
DKEFS switching: accuracy	40	40
DKEFS color/word inhibition	20	37
DKEFS color/word inhibition switch	20	40
DKEFS word context test	40	53

*Language functioning*		

Boston naming test	N/A	N/A
Category fluency (animal naming)	37	53
DKEFS letter fluency	37	53
BDAE complex ideational material	n/a	N/A
DKEFS word context test	40	53
DKEFS category fluency	See above	53

*Memory functioning*		

WMS-IV logical memory I	23	40
WMS-IV logical memory II	23	33
WMS-IV verbal paired associates I	33	43
WMS-IV verbal paired associates II	23	47
WMS-IV visual reproduction I	37	43
WMS-IV visual reproduction II	27	50
WMS-IV designs I	43	N/A
WMS-IV designs II	40	N/A
